# Implant Size and Fixation Mode Strongly Influence Tissue Reactions in the CNS

**DOI:** 10.1371/journal.pone.0016267

**Published:** 2011-01-26

**Authors:** Jonas Thelin, Henrik Jörntell, Elia Psouni, Martin Garwicz, Jens Schouenborg, Nils Danielsen, Cecilia Eriksson Linsmeier

**Affiliations:** 1 Neuronano Research Centre, Lund University, Lund, Sweden; 2 Center for Psychology, Kristianstad University, Kristianstad, Sweden; 3 Department of Psychology, Lund University, Lund, Sweden; Tokyo Medical and Dental University, Japan

## Abstract

The function of chronic brain machine interfaces depends on stable electrical contact between neurons and electrodes. A key step in the development of interfaces is therefore to identify implant configurations that minimize adverse long-term tissue reactions. To this end, we here characterized the separate and combined effects of implant size and fixation mode at 6 and 12 weeks post implantation in rat (n = 24) cerebral cortex. Neurons and activated microglia and astrocytes were visualized using NeuN, ED1 and GFAP immunofluorescence microscopy, respectively. The contributions of individual experimental variables to the tissue response were quantified. Implants tethered to the skull caused larger tissue reactions than un-tethered implants. Small diameter (50 µm) implants elicited smaller tissue reactions and resulted in the survival of larger numbers of neurons than did large diameter (200 µm) implants. In addition, tethering resulted in an oval-shaped cavity, with a cross-section area larger than that of the implant itself, and in marked changes in morphology and organization of neurons in the region closest to the tissue interface. Most importantly, for implants that were both large diameter and tethered, glia activation was still ongoing 12 weeks after implantation, as indicated by an increase in GFAP staining between week 6 and 12, while this pattern was not observed for un-tethered, small diameter implants. Our findings therefore clearly indicate that the combined small diameter, un-tethered implants cause the smallest tissue reactions.

## Introduction

Brain-machine interfaces (BMI's) have a wide range of applications in both clinical practice and experimental research. The possibility to record from, or stimulate, central nervous tissue over long periods of time provides a unique basis both for diagnosing and treating patients with neurodegenerative or psychiatric disorders and for characterizing fundamental neural mechanisms in animal models [1], [2], [3], [4]. At the core of the typical BMI is the electrode or electrode array, implanted chronically in the central nervous system. Inevitably, the implantation procedure is associated with a certain amount of local tissue damage and the implant itself subsequently elicits both acute and chronic reactions in the surrounding tissue [5], [6]. Histologically, these reactions are manifested as a zone of activated astrocytes surrounding a core of activated microglia adjacent to the implant surface. Within this zone of gliosis or reactive capsule, a reduction of neuronal density has been described [7], [8]. These tissue responses may have detrimental effects on the long term function of the electrode.

The present study is based on the assumption that there are several factors underlying the long-term success of an implanted electrode. Besides electrode design and recording properties one of the key factors determining long-term function of neural interfaces is the functional distance between neurons and recording/stimulation sites and the stability of this distance over time. If the functional distance is increased, either by loss of neurons in close vicinity to the electrode or by a progressively growing glial capsule, the function of the electrode will be compromised. The formation of a glial capsule may also *per se* jeopardize electrode function by increasing the electrical resistance/impedance. These changes must therefore be minimized to ensure the long-term high quality recordings necessary for analysis of processes such as memory formation, or maintained stimulation efficacy necessary for obtaining adequate and stable therapeutic effects in the clinical context.

In previous studies, one of the factors deemed to be important for minimizing the unwanted tissue reactions has been the use of un-tethered, rather than tethered, electrodes. The rationale for this approach is to minimize the motion between electrode and brain tissue caused by the normal movements of the brain within the skull cavity due to forces induced by respiration and circulation [9]. A tethered design may also allow invasion of unwanted cells such as meningeal fibroblasts into the brain tissue [7], [9]. However, although there seems to be consensus that un-tethered implants elicit a smaller tissue reaction than tethered ones, it is still unclear if the actual neuronal numbers differ between the two fixation modes. In a quantitative immunohistochemical study using rather large implants [7] it was shown that tethered implants induced a significantly larger astrocytic and microglial response at 4 weeks, compared to non-tethered ones. This report was followed up by a quantitative study comparing tethered and un-tethered silicon microelectrodes for 1–4 weeks [9]. Tethered electrodes induced a more severe astrocytic and microglial response. Immunohistochemical staining for neurofilaments showed a reduced expression for tethered electrodes, suggesting a reduced neuronal density, but no actual cell counts were presented [9]. Although this research group had previously demonstrated that tethered electrode implantation induced a reduced neuronal density using cell counting methods (about 40% and most obvious within a 100 µm radius from the implant) [5], the evaluation period was rather short (2–4 weeks) [5]. In a recent study it was shown that stainless micro-wires implanted and tethered to the skull induced a persistent inflammation over a 12 week evaluation period, but the reactive gliosis and the reduction in neuronal density within a 50 µm radius from the implant were not progressive [6]. Taken together, these findings underscore the need for a study systematically comparing the two fixation modes by evaluating the overall elicited tissue reactions and specifically addressing in quantitative terms the issue of neuronal numbers close to the implants over rather long evaluation periods.

Another factor of importance for the tissue reactions elicited may be the size of the implant, but previous findings are contradictory: While some studies have suggested that long-term tissue responses (more than 2 weeks) are independent of implant size [10], others have reported that smaller diameter implants induce a smaller astrocytic reaction than larger devices [11]. In a non-quantitative immunohistochemical study three different implants with various shapes and sizes were compared for up to 12 weeks after implantation [10]. It was claimed that device size was the major factor to early tissue responses and responses after 4 weeks were similar for all devices. The question of size was specifically addressed by Stice *et. al.* (2007) [11]. This study demonstrated that GFAP expression was significantly smaller for 12 µm diameter implants as compared to 25 µm implants at the longer evaluation period, which was 4 weeks. Notably, the relative importance of implant size and fixation mode, and the possible interplay between the two factors have not been established or quantified.

Electrodes implanted in humans are expected to function for a very long time, in some cases nearly a whole lifetime and human and primate studies have shown that electrodes can function for several years [12], [13]. In rabbits, 6 month follow up periods have been used [8]. Most experimental rat studies uses rather short evaluation periods with an emphasis on the evaluation periods of 2–4 weeks [5], [7], [9], [10], [11], even though some studies have follow up periods up to 16 weeks [14]. Since changes over time are important to consider when systematically evaluating how the characteristics of the implant influence the tissue, we here focused on establishing and quantifying the relative importance and potential interactions between implant size and fixation modes over time. To this end, we used quantitative immunohistochemical methods to compare the tissue reactions caused by 50 µm and 200 µm diameter implants, either tethered or un-tethered to the skull, focusing especially on the zone within a 50 µm radius from the implant-tissue border and using an evaluation period of 12 weeks. The 50 µm distance is of immediate relevance to neurophysiological recordings since distances over which spiking activity of individual neurons can be followed rarely exceeds 50 µm [15]. We deliberately chose to use rounded implants to avoid the tissue reactions related to sharp edges and corners and thereby also avoiding the problem of compensating for the corners when calculating neuronal density [8].

## Materials and Methods

### Animals and surgery

The study was approved by the Malmo/Lund Animal Ethics Committee on Animal Experiments (permit number M143-08). We used a total of 24 adult female Sprague-Dawley rats (Taconic, Denmark) weighing approximately 220 g at the beginning of the experiment. All the animals were housed under a 12 h light/dark cycle with free access to water and food. For surgery, the rats were deeply anaesthetized by intraperitoneal injections of a mixture of Fentanyl (50 mg/mL) and Domitor vet 1 mg/mL (medetomidin hydrochloride) as previously described [16]. After surgery the animals received subcutaneous injections of 1 mL/kg body weight of a mixture of Antisedan vet 5 mg/mL (antipamezole hydrochloride) and sterile water. This injection serves as an antidote to the anesthesia. At the same time the animals received analgesia subcutaneously.

### Implants

**Figure 1:**
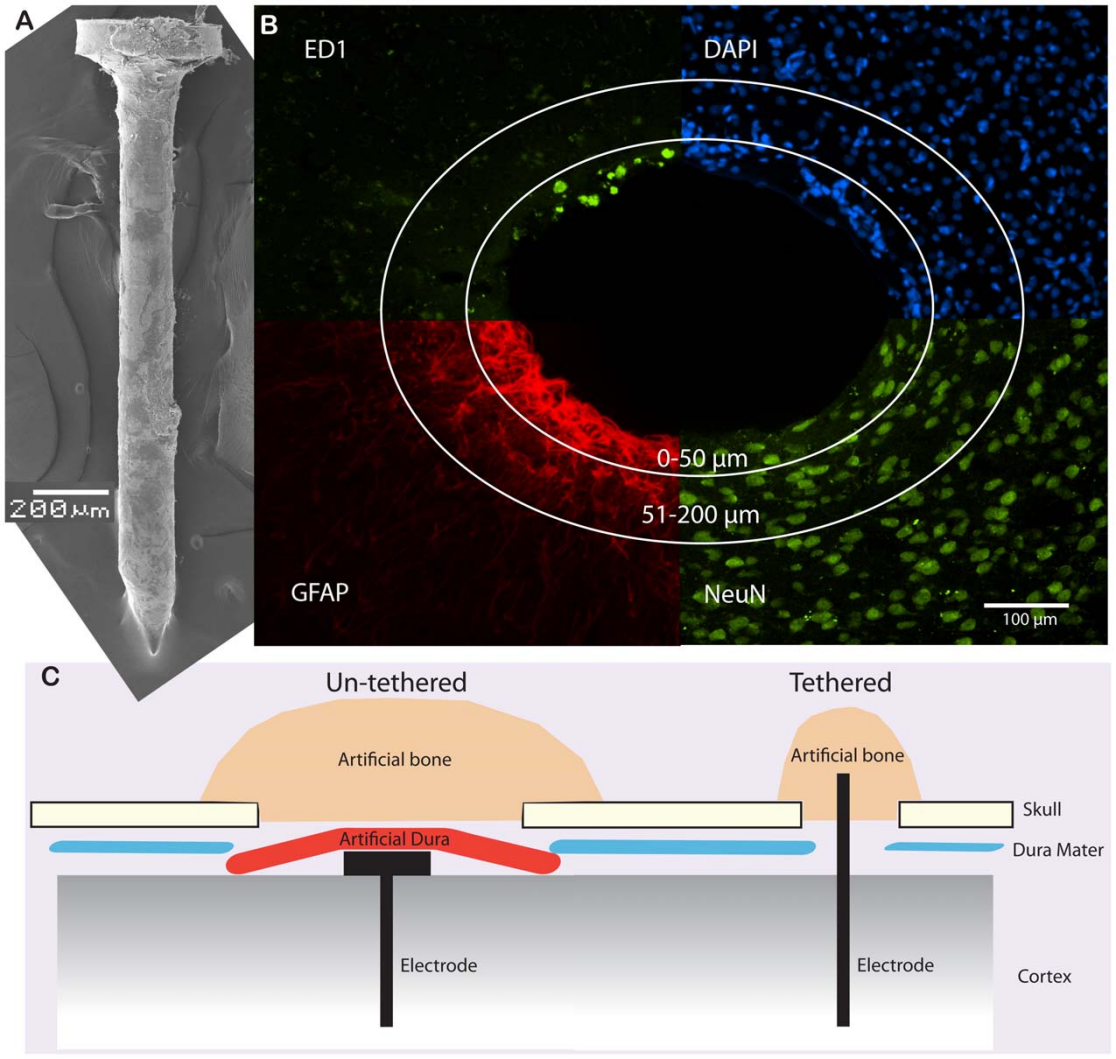
An overview of methods used in this study. **A**. A SEM-picture of a 200 µm stainless steel probe used in the un-tethered fixation model. **B**. The picture is a montage of the four different staining used, ED1 (activated microglia), DAPI (all cell bodies), GFAP (activated astroglia) and NeuN (neural cell bodies). The circles illustrate the two different regions of interest that were analyzed. **C**. The two different implantation techniques are illustrated. The un-tethered technique was ensured by adding artificial dura on top of the implant, separating the implant from the skull. In order to seal the opening in the skull, tissue friendly artificial bone was applied.

Since the aim was to investigate the impact of size and fixation mode of the implant on the tissue response, we compared a 200 µm to a 50 µm implant diameter, and a tethered to an un-tethered fixation mode. To this end, we manufactured in our engineering workshop four different types of stainless steel (DIN 1.4401) sham electrodes, i.e. containing no electronics. Two types, both 1.8 mm long, were made for an un-tethered design with a nail configuration, at diameters: (a) ∅ 50 µm, nail head 150 µm in diameter and (b) ∅ 200 µm (see figure 1A), nail head 500 µm. Two types with different diameters (50 µm vs. 200 µm), both 3 mm long, were made for a tethered design with no nail head. All the implants were sterilized in ethanol (70%) overnight. The use of these four implants provides data for a full factorial design for the study (implant diameter: 200 µm vs. 50 µm, fixation mode: tethered vs. un-tethered), which allows the assessment of effects of fixation mode and implant size independently of each other, as well as the evaluation of the different implant configurations.

### Implantation procedure

All procedures involving animal surgery were performed using sterile techniques. All animals received an un-tethered implant on one side and a tethered implant on the contra lateral side of the cerebral cortex. For implantation, animals were deeply anesthetized as described above, prepared for surgery by shaving the head and placed in a stereotactic frame (KOPF Instruments, USA) set under a stereomicroscope (Leica Microsystems, M651, Germany). The surgical area of the scalp was disinfected using 70% ethanol, and a 3 cm midline incision was made to expose the skull. Thereafter, the tissue attached to the skull was removed and blood was cleansed away. For all implant types a 2 mm diameter burr hole was drilled on both sides of the midline (coordinates: 0.5 mm rostral from the bregma and ±2.5 mm lateral). The burr hole was rinsed with sterile phosphate buffered saline (PBS). The dura mater was cut open with forceps and a fine pair of scissors and a hydraulic micromanipulator (KOPF Instruments, USA) was used to lower the implants into the brain at a rate of 10 µm/sec. The implants were fastened to the micromanipulators using gelatine as glue, which is fast-dissolving in contact with water (cerebrospinal fluid, blood, PBS) and body heat. When the implants were in place and the gelatine was dissolved the micromanipulator was withdrawn. This new method ensured minimal or no movement of the implants when retracting the micromanipulator. When implanting the un-tethered implants they were inserted 1.8 mm into the cortex resulting in that the nail head was placed on the cortex surface. There was no major bleeding observed during the insertion of the electrodes. After implantation, the nail head was covered with an artificial dura mater (Tissudura, Baxter, Germany) to minimize the risk of implant attachment to the skull and surrounding tissue. The hole in the skull was closed using a fast hardening bone graft substitute (Stratec Medical, Germany). The tethered implants were implanted approximately 1.8 mm into the cortex and fastened in the bone using the same bone graft substitute as used for the un-tethered. The implantation procedures are depicted in figure 1C. Finally, skin was closed using surgical clips (Michell, 7.5×1.75). Twelve animals for the study of each implantation time period were used (6 and 12 weeks).

### Tissue fixation and sectioning

After 6 and 12 weeks respectively, animals were anaesthetized with an overdose of pentobarbital (i.p.) and transcardially perfused with 150–200 ml ice-cold 0.1 M phosphate buffer (PB), followed by 4% paraformaldehyde (PFA) in 0.1 M (PB). After the brains were removed and postfixed at 4°C overnight in fixative, they were soaked overnight in 0.1 M PB containing 25% sucrose for cryopreservation and subsequently sectioned in the horizontal plane at 30 µm, using a sliding knife freezing microtome (Microm, Germany). In order to ensure that the same area (depth) was analyzed between each individual rat all brains were sectioned in the horizontal plane, with a section thickness of 30 µm. By keeping track of each individual section it was possible to define the area (depth) of the analysis. Great care was taken to assure that the sections were perpendicular to the implant direction. However, prior to sectioning, when the brains were frozen on the microtome, both un-tethered and tethered implants were carefully explanted, labelled and saved in PBS for further evaluation. The implants were investigated in a sweep electron microscope and there was no significant tissue adherence on any of the implants (see Figure 1A).

### Antibodies

The primary antibodies used were rabbit polyclonal antibodies recognising Glial Fibrillary Acidic Protein (GFAP, an astrocytic cytoskeleton protein, 1∶5000, Dako, Denmark), and mouse monoclonal antibodies recognising either CD68/ED1 (expressed by activated microglia, 1∶250, AbD Serotec, UK) or NeuN (expressed on neuronal cell nuclei, 1∶100, Chemicon, USA). Thereafter, sections were incubated in DAPI (cell nuclei marker, 1∶1000, Invitrogen, USA), Alexa488-conjugated antibodies for mouse IgG and Alexa594-conjugated antibodies for rabbit IgG (1∶100, Invitrogen, USA).

### Immunohistochemistry

The gliosis (i.e. capsule thickness), the recruitment of microglial cells and the distances between the implant surfaces to the nerve cells were evaluated using free-floating immunohistochemical techniques. Hence, following rinses of sections in potassium phosphate buffered saline (KPBS, 0.02 M, pH 7) and preincubation in a mixture of 5% normal serum and 0.25% Triton X-100 (Sigma, Germany) in 0.02 M KPBS, the sections were reacted with the primary antibodies (see above) overnight at room temperature. After repeated rinses in KPBS, they were further incubated with secondary antibodies (see above) (2 h, dark, room temperature) and rinsed in KPBS. The sections were then mounted onto chrome alum coated slides and cover slipped with Vectashield Hardset mounting media (Vector, USA) or PVA/DABCO (FLUKA, Switzerland). For all the different antibody protocols, controls with omission of primary antibodies were negative.

### Image processing

All histological fluorescence images were obtained using a DS-2Mv Digital camera (Nikon Instruments, Japan), mounted on a Nikon Eclipse 80i microscope with a 10× objective (Nikon Instruments, Japan). The images were acquired and analyzed using the NIS-Elements BR software 3.05 (NIS-Elements, Nikon Instruments, Japan). In contrast to other studies the regions of interest (ROIs) were set at 0–50 µm and 51–200 µm from the rim of the artifact caused by the implants or circumferenting the center of the wound when no clear hole was found at the distant tip. The 0–50 ROI was chosen in order to investigate the number of neurons present within recordable distance from the implant (see figure 1B). Because of the variability of the specificity of the different markers for their respective antigens, the thresholds were set at individual levels for each marker, corresponding to differences in the contrast between unspecific background staining and positively stained antigens. The larger the contrast between the background and positively labelled tissues, the higher the threshold was set. Thus, the intensity thresholds for GFAP and ED1 and DAPI were set at 3 times of background intensity. Total area and the area containing pixels above the intensity threshold were measured within each ROI [17], [18]. The results were expressed as the fraction between area above threshold and total area of the ROI. Sections from the middle of the implant tract, i.e. at an approximate depth of 750–1050 µm, were chosen for imaging. The section at the largest depth that still showed a clear wound from the implant was also imaged. The neurons were stained with NeuN and manually counted on microscopic images by the same person using the NIS-Elements BR software 3.05, in a blinded manner. All NeuN positive cells were counted from the rim of the artifact to the 200 µm marker (figure 1B). The design of the study allowed for analyzing relative differences regarding neuronal densities(presented as neurons/µm^2^) between different electrode configurations [19].

### Cavity shapes and altered nerve cell morphology

In order to obtain a quantitative approximation of the altered neuron morphology we defined two prototypic sections (Figure 5), one as an example of altered organisation of nerve cells and one as an example of no alteration, and asked three untrained individuals, blind to the aims of our study or to the implant types used, to separate 36 sections into ‘altered’ and ‘non-altered’ nerve-cell organization, respectively.

**Figure 2:**
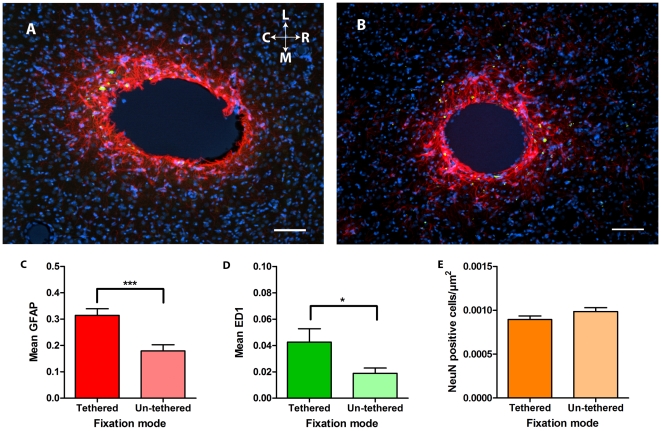
Example pictures and results for the two different time points. **A**. Tissue reaction to a 200 µm tethered implant after twelve weeks. Sections are immunohistochemically labelled for GFAP (red), ED1 (green) and DAPI (blue). Scale bar lower right is 100 µm. The orientation of the picture is indicated as rostral (R) caudal (C) medial (M) and lateral (L). **B**. Tissue reaction to a 200 µm un-tethered implant after twelve weeks. Sections are immunohistochemically labelled for GFAP (red), ED1 (green) and DAPI (blue). Scale bar lower right is 100 µm. **C**. Quantified GFAP density surrounding (0–200 µm) the implants with respect to fixation mode. The columns indicate the mean and bars show the standard error of the mean. ***p<0.001. **D**. Quantified ED1 density surrounding (0–200 µm) the implants with respect to fixation mode. The columns indicate the mean and bars show the standard error of the mean. *p<0.05. **E**. Number of neurons surrounding (0–200 µm) the implants with respect to fixation mode. The columns indicate the mean and bars show the standard error of the mean.

### Design and Statistical analysis

To evaluate the evoked tissue reactions of the different implants we used GFAP defined as activated astrocytes, ED1 defined as activated microglia and NeuN defined as neurons (Figure 1B). In a first round of analysis, linear 2x2x2x2x2 models (MANOVA/ANOVA) were used to evaluate independent quantitative effects of implant *Fixation mode* (tethered vs. un-tethered), implant *Diameter* (50 µm vs. 200 µm), *Time-point* after implantation (6 vs. 12 weeks), *Distance* from implant (0–50 µm vs. 50–200 µm) and *Depth* of section (shank region vs. tip region of implant), on GFAP, ED1and NeuN values seen together, and separately. In a second round, we replaced implant *Fixation mode* and *Diameter* with *Implant type* (50 µm-tethered vs. 50 µm-un-tethered vs. 200 µm-tethered vs. 200 µm un-tethered) and repeated the analysis (4x2x2x2 multifactor ANOVA). The Bonferroni test was used for post-hoc comparisons. For all the analyses, statistical significance was defined at the 5% level. Partial *η* ^2^ are reported as estimates of size effects. All statistical analysis was performed in PASW (version 18.0). In a third step, further addressing outcomes of implantation that may be expected to influence recording or stimulation characteristics, the shapes of neurons surrounding the implant cavity and the size and shape of the cavity itself were examined. In order to compare the different implants effects on the tissue, all data used in the comparison of cavity sizes were normalized to the actual size of each implant. A nonparametric t-test (Mann-Whitney) was used for statistical evaluation of cavity size (GraphPad Prism 5.02). Significant differences were assumed at the level of p<0.05.

## Results

The analysis was divided into three steps. First, in order to establish the relative importance of individual experimental variables on the tissue reaction including number of neurons, effects of implant *fixation mode* and *diameter*, *time-point* after implantation and *distance* from implant were analyzed. Second, in order to find the optimal implant configuration in this study, effects of the four actual implant types – tethered or un-tethered with small or large diameter, respectively – were compared. In a third step, further addressing outcomes of the implantation that may be expected to influence recording or stimulation characteristics, the shapes of neurons surrounding the implant cavity and the size and shape of the cavity itself were examined.

### Effects of individual experimental variables

Multivariate analysis of variance (MANOVA) was carried out to assess possible statistically significant differences across the levels of our independent variables for a linear combination of our dependent variables (GFAP, NeuN, ED1). Indeed, there were significant multivariate main effects of both *fixation mode* (*F*_(3, 254)_  = 14.85, *p*<.0001, partial *η* ^2^ = .15), *diameter* (*F*_(3, 254)_  = 48.68, *p*<.0001, partial *η* ^2^ = .37), and *time-point* (*F*_(3, 254)_  = 18.06, *p*<.0001, partial *η* ^2^ = .17) but also *distance* (*F*_(3, 254)_  = 47.48, *p*<.0001, partial *η* ^2^ = .36) on GFAP; ED1 and NeuN linearly combined (Pillai's trace method was used, as the most robust alternative in such analysis, see Tabachnick & Fidell, 2007) [20].

#### Fixation mode (un-tethered vs. tethered)

Independently of electrode diameter, time-point after implantation, distance and depth of section, the un-tethered fixation mode resulted in significantly lower GFAP (*F*_(1, 256)_  = 38.24, *p*<.0001, partial *η* ^2^ = .13) and ED1 (*F*_(1, 256)_  = 4.52, *p*<.05, partial *η* ^2^ = .02) than did tethered. This means that un-tethered fixation mode resulted in fewer activated astrocytes and microglia. Fixation mode did not affect the density of neurons (Figure 2 and Table 1).

**Figure 3:**
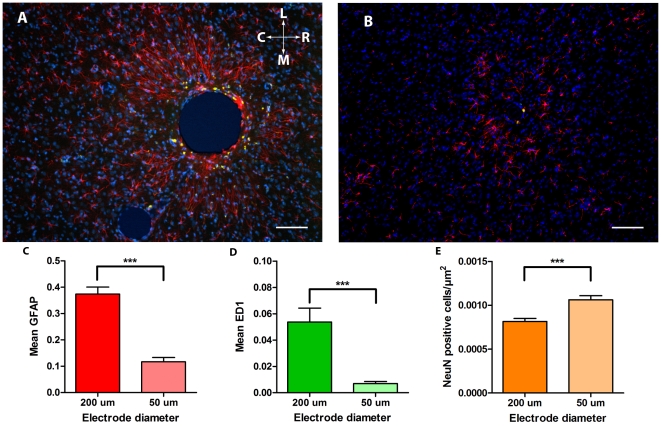
Example pictures and results for the two different electrode sizes. **A**. Tissue reaction to a 200 µm un-tethered implant after twelve weeks. Sections are immunohistochemically labelled for GFAP (red), ED1 (green) and DAPI (blue). Scale bar lower right is 100 µm. The orientation of the picture is indicated as rostral (R) caudal (C) medial (M) and lateral (L). **B**. Tissue reaction to a 50 µm un-tethered implant after twelve weeks. Sections are immunohistochemically labelled for GFAP (red), ED1 (green) and DAPI (blue). Scale bar lower right is 100 µm. ***p<0.001. **C**. Quantified GFAP density surrounding (0–200 µm) the implants with respect to diameter. The columns indicate the mean and bars show the standard error of the mean. ***p<0.001. **D**. Quantified ED1 density surrounding (0–200 µm) the implants with respect to diameter. The columns indicate the mean and bars show the standard error of the mean. ***p<0.001. **E**. Number of neurons surrounding (0–200 µm) the implants with respect to diameter. The columns indicate the mean and bars show the standard error of the mean. ***p<0.001.

**Table 1:** Results from the multivariate analysis of variance.

Variables	Group	GFAP	ED1	NeuN
Fixation mode	Tethered/Untethered	***	*	ns
Diameter size	200 µm/50 µm	***	***	***
Time point	6 weeks/12 weeks	***	*	ns
Distance from electrode	0–50 µm/51–200 µm	***	***	***

Results from the multivariate analysis. ns =  no significant,

*p<0.05,

*** p<0.001.

#### Diameter (50 µm vs. 200 µm)

Independently of fixation mode, time-point after implantation, distance and depth of section, the small diameter electrodes (50 µm) resulted in significantly lower GFAP (*F*_(1, 256)_  = 110.7, *p*<.0001, partial *η* ^2^ = .30) and ED1 (*F*_(1, 256)_  = 19.69, *p*<.0001, partial *η* ^2^ = .07) and greater density of neurons (*F*_(1, 256)_  = 20.91, *p*<.0001, partial *η* ^2^ = .08) than did the large diameter. This means that smaller diameter electrodes resulted in fewer activated astrocytes and microglia and more NeuN positive cells (Figure 3 and Table 1).

**Figure 4:**
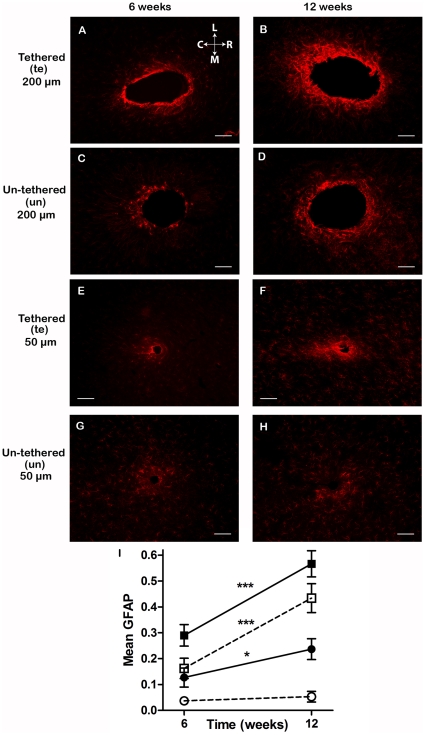
Effects of each actual electrode type separately. Comparison of the GFAP-staining, spatial and temporal reactivity surrounding un-tethered and tethered implants of both 200 µm and 50 µm in diameter. Representative images of horizontal sections perpendicular to the implantation tract in the cortex of adult rats receiving 200 µm tethered (**A,B**)and un-tethered (**C,D**) or 50 µm tethered,(**E,F**)and un-tethered (**G,H**) implants at 6 weeks (**A,C,E,G**) and 12 weeks (**B,D,F,H**) after implantation, illustrating an elevated GFAP immunoreactivity in brain tissue surrounding the implants. The tissue sections shown were from the mid cerebral cortex at a depth of approximately 750–1050 µm. Scale bar 100 µm. The orientation of the picture is indicated as rostral (R) caudal (C) medial (M) and lateral (L).**I**. Graph showing the mean GFAP response over time for the four different implant types irrespective of distance from the implant. Symbols indicate mean and the bars show standard error of the mean. ▪ = 200 µm tethered, □ = 200 µm un-tethered, • = 50 µm tethered, ○ = 50 µm un-tethered. * p<0.05, *** p<0.001.

#### Time-point after implantation (6 vs. 12 weeks)

Independently of electrode fixation mode or diameter, distance and depth of section, there were increased values of GFAP(*F*_(1, 256)_  = 51.45, *p*<.0001, partial *η* ^2^ = .17) and decreased values of ED1 (*F*_(1, 256)_  = 4.31, *p*<.05, partial *η* ^2^ = .02) at 12 weeks compared to 6 weeks post implantation, indicating an increase of activated astrocytes combined with a decrease in activated microglia after 12 weeks (see Table 1).

#### Distance from electrode (0–50 µm vs. 50–200 µm)

Independently of electrode fixation mode or diameter, time-point and depth of section, there were higher values of GFAP (*F*_(1, 256)_  = 79.7, *p*<.0001, partial *η* ^2^ = .24) and ED1 (*F*_(1, 256)_  = 23.62, *p*<.0001, partial *η* ^2^ = .08) closer to the electrode, and fewer NeuN positive cells (*F*_(1, 256)_  = 50.22, *p*<.0001, partial *η* ^2^ = .17) closer to the electrode (see Table 1).

### Optimal electrode configuration

Considering the robust effects of both electrode fixation mode and diameter on the tissue reaction we proceeded, in the second round of analysis, to test the effects of each actual electrode type separately. We found robust multivariate main effects (Pillai's trace) of *electrode type* (50 µm tethered vs. 50 µm un-tetheredvs. 200 µm tethered vs. 200 µm un-tethered: *F*_(9, 768)_  = 14.90, *p*<.0001, partial *η* ^2^ = .15), *time-point* (*F*_(3, 254)_  = 18.06, *p*<.0001, partial *η* ^2^ = .18), *distance* (*F*_(3, 254)_  = 47.48, *p*<.0001, partial *η* ^2^ = .36) on GFAP, ED1 and NeuN together. The univariate effects of electrode type were significant for GFAP (*F*_(3, 256)_  = 51.14, *p*<.0001, partial *η* ^2^ = .38; Figure 4), where progressively less activated astrocytes were found as one advanced from a 200 µm tethered electrode to a 200 µm un-tethered, and further to a 50 µm tethered, and finally a 50 µm un-tethered electrode, where the least number of activated astrocytes was found (all Bonferoni post-hoc comparisons significant at p<.0001). Univariate effects of electrode type were also significant for ED1 (*F*_(3, 256)_  = 9.05, *p*<.0001, partial *η* ^2^ = .10) where the 200 µm tethered electrode produced significantly higher values, indicating more activated microglia, than both the 50 µm un-tethered and the 50 µm tethered (p<.0001), as well as for NeuN (*F*_(3, 256)_  = 8.32, *p*<.0001, partial *η* ^2^ = .09), where the 200 µm tethered electrode produced significantly lower values, indicating less positive neurons, than both the 50 µm un-tethered and the 50 µm tethered (p<.0001).

**Figure 5:**
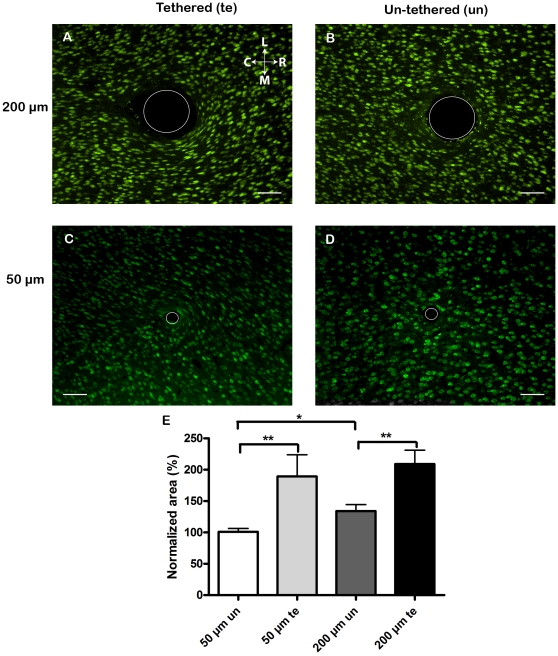
Nerve cell patterns and cavity shapes. Representative images of horizontal sections perpendicular to the implantation tract in the cortex of adult rats, stained with NeuN showing the neural cell bodies. Note the orientation of the cells close to the tethered implants (**A,C**) in comparison with the un-tethered implants (**B,D**). White circle indicate the actual size of the implant. Scale bar 100 µm. The orientation of the picture is indicated as rostral (R) caudal (C) medial (M) and lateral (L). **E**. The normalized area of the cavity made by the implants, for the 50 µm and the 200 µm implants. Columns indicate mean and bars the standard error of the mean. (un) un-tethered, (te) tethered. *p<0.05, ** p<0.01.

Electrode type interacted with *time-point* (*F*_(3, 256)_  = 8.18, *p*<.0001, partial *η* ^2^ = .09; Figure 4), and *distance* on GFAP values (*F*_(3, 256)_  = 7.81, *p*<.0001, partial *η* ^2^ = .08 – Figure 4) and with *distance* on ED1 (*F*_(3, 256)_  = 5.79, *p*<.001, partial *η* ^2^ = .06). There was also an interaction of electrode type with Depth of section on NeuN values (*F*_(3, 256)_  = 3.03, *p*<.05, partial *η* ^2^ = .03), as 50 µm diameter electrodes resulted in significantly more positive NeuN cells nearer the tip compared to the middle of the electrode, while this was not the case for 200 µm diameter electrodes. Furthermore, some of the 50 µm un-tethered electrodes, exhibited a diffuse GFAP staining (see Figure 4H) 12 weeks after implantation. The GFAP staining appeared relocated and formed a diffuse band at some distance from the electrode, in contrast to the staining closely surrounding the other electrode types.

### Nerve cell patterns and cavity shapes

A previously unreported finding was that there appeared to be an alteration in the shapes and organisation of nerve cells (flattened neurons in a whirl-like pattern close to the implant) surrounding the tethered implants (see Figure 5). Untrained persons were asked to separate pictures of altered and unaltered neuron morphology. This resulted in a separation of section related to tethered implants and un-tethered implants with a mean accuracy of 88%, indicating that clearly visible patterns were formed by the tethered implants.

Also the shapes and sizes of the cavities made by the implants demonstrated different characteristic properties depending on implant fixation mode. The un-tethered implants typically made a round cavity, whereas the tethered implants gave rise to an elongated, oval cavity with the long axis in the rostral to caudal plane. In order to facilitate comparisons between the two different diameters of implants used, we normalized the size of the cavity relative to the actual implant size. Since there was no significant difference in cavity size when comparing 6 weeks and 12 weeks after implantation, we investigated fixation mode regardless of time point. When quantifying the area of the different cavities we found a significant (p<0.01) enlargement in relation to actual implant size made by tethered implants regardless of diameter (Figure 5E). The rostral-to-caudal edge-to-edge distance of the oval shaped cavities was significantly (p<0.001) longer than the medial-to-lateral one for both small and large diameter tethered implants. Furthermore, there was a trend (not significant p = 0.0756) towards a larger normalized rostral-to-caudal edge-to-edge distance for the un-tethered 200 µm implant compared to the un-tethered 50 µm implant. Interestingly, when comparing instead normalized areas, the un-tethered 200 µm implant made a significantly (p<0.05) larger cavity compared to the un-tethered 50 µm implant (Figure 5E). There was no significant difference for any of the implant types when measuring the edge-to-edge distance in the medial-to-lateral axis.

## Discussion

Finding the configuration of electrode implants that evokes minimal tissue reaction is a key issue in the development of future brain machine interfaces. The present study focused on basic properties of implanted electrodes, and could demonstrate that both size and fixation mode of the implant influence tissue trauma and healing. Small diameter electrodes elicited much smaller tissue reactions including preservation of a relatively greater number of neurons than larger diameter ones. Importantly, tethering the electrodes to the skull not only caused a larger tissue response but, in addition, resulted both in an seemingly irreversible oval-shaped cavity, with a cross-section area larger than that of the actual implant itself, and in marked changes in the morphology and organization of neurons in the region closest to the tissue interface. Such changes in the innermost region were not seen with un-tethered electrodes. The present study therefore clearly demonstrates that small diameter un-tethered electrodes cause the smallest tissue reactions and tissue deformation.

### Factors influencing durability of electrode implants

In order to successfully record from implanted electrodes, a sufficient number of nearby neurons must survive the implantation procedure and the functional distance between neurons and the electrode recording site cannot be too long. The damage to the central nervous system caused by implanting an electrode induces a series of events. The main cells involved are astrocytes, activated microglia and oligodendrocyte precursors [21]. In previous analyses, focus has been on the glial scar, especially the astrocytic part, since glial scarring will jeopardize the function of neural electrodes [22], [23]. Glial activation following an implantation is important for the function of the neural interface in several ways. In the acute phase, microglia from the surrounding tissue and macrophages from the bloodstream invade the injured region and astrocytes are activated, which is presumably important for the healing process. In the present study we also addressed the long-term phase, since chronic reactions may jeopardize the function of the electrode, and therefore the durability of the implant, in a number of ways. First, the ongoing activation of microglia and astrocytes may eventually cause a ‘scar’ around the electrodes and thereby displace the neurons to be recorded from or to be stimulated. This will decrease the signal to noise ratio in a recording electrode and increase the stimulus threshold in a stimulating electrode. Second, the activated microglia may attack the electrode implant itself causing malfunction of the implant over time. Thirdly, the chronic inflammation elicited by the electrode may lead to neuronal cell loss and subsequent recording failure [5].

### Electrode diameter and evaluation time

The experimental variable that most strongly influenced the tissue response in the present study was the diameter of the implant. Large diameter implants elicited the largest tissue responses and these responses were ongoing for at least 12 weeks. Implantation of a foreign material will always induce some type of cellular or tissue response. The important matter is that this response is small enough to allow recording of nerve signals and that the tissue reaction is not progressing over time, thereby jeopardizing long-term function. Even very small structures produce tissue responses [17]. In a recent study, the tissue responses to silica coated nanowires (120 nm in diameter and 2 um long) injected in the brain was studied and revealed typical glial responses [17]. The glial response 12 weeks after implantation was diminishing but still detectable. Activated microglial cells that had engulfed the nanowires were also present. Even longer follow up times are probably needed, in order to fully evaluate just how critical implant size is.

### Un-tethered versus tethered electrodes

Our data clearly support previous findings that the brain tissue response is increased when implanted devices are tethered to the skull [9]. However, perhaps the most dramatic finding in this study was that tethered electrodes were surrounded by an oval-shaped cavity with an area extending that of the original implant, thus causing an increased functional distance between the electrode and tissue. This previously probably overlooked effect was most pronounced in the anterio-posterior axis, presumably reflecting that movements of the brain relative to the skull during daily life are more pronounced than movements in the medial–lateral axis [24]. Whether or not the cavity around the implanted electrode is the result of loss or displacement of tissue is not known, but is evident that a relatively larger tissue cavity will affect the recording properties of an electrode. More importantly, increased functional distance between neurons and recording sites will affect implant function negatively. Another negative factor is the presence of extracellular fluid between the implant surface and the tissue border. Previous research has shown that soft tissue implants are surrounded by a fluid space [25], [26]. However, such a fluidic zone will short-circuit the extracellular currents produced by active nearby neurons and thereby dramatically reduce the signals recorded. If the notion is correct, that the fluid zone results from movements between the tethered electrode and tissue, this finding also indicates that the stability of the recordings will be impaired by the electrode being tethered to the skull.

The oval shaped cavities and the de-arranged morphology for neurons surrounding tethered electrodes are one of the main finding in this study, although not fully quantified here. Cell bodies of neurons close to the tissue interface appeared to attain a flattened morphology and the general cellular pattern was changed around tethered electrodes. In contrast, neurons with apparently normal morphology were abundant in the inner ROI of the small diameter un-tethered electrodes. The functional implications of these findings can only be speculative at this point in time and require further investigation.

Note that the ROIs defined in this study, while generally conforming to those of other groups [5], [9], are related to the surface of the cavity. Hence, in case of a developed fluid zone around the tethered electrode, the inner border of the innermost ROIs starts at a distance from the electrodes. The resulting ROIs of tethered and un-tethered electrodes will therefore differ in their distance from the electrode. As the tissue response generally declined at a distance from the electrodes, the fact that the glial response was larger around the tethered electrodes therefore masks an even larger difference between tethered and un-tethered electrodes. Moreover, the finding of altered morphology and organization of neurons in the inner ROIs of tethered electrodes is in accordance with larger glial response in this region.

### Future perspective on electrode design

Today there are basically three types of devices being used for recording neural signals from the motor cortex, the Michigan and Utah electrodes, and insulated micro-wires. Michigan electrodes are silicon or polymer based needles with several surface electrode sites along the shaft [27], [28]. These needles can also be arranged in an array format to increase the number of electrode sites [29], [30]. Utah electrodes are silicon needles arranged in a two-dimensional format, typically 10×10, where the tip of each needle is the electrode site [13], [31], [32], [33], [34]. Insulated wires can be manually arranged in a three-dimensional pattern [12], [35], [36], [37].

In the present study we deliberately avoided issues of different electrode designs and focused instead on two basic electrode characteristics, electrode diameter and fixation mode. We also chose not to use functioning electrodes but rather a principle for a singular electrode made of a non-functioning material, stainless steel, in order to try to define general principles for electrode implantation into the central nervous system. Our results strongly suggest that in order to ensure a close relationship between neurons and recording sites, it is useful to minimize electrode diameter until a functionally reliable un-tethered electrode has been developed. The effects of flexible electrodes, either tethered or un-tethered is still unknown.
